# Electroosmotic Flow in a Rough Nanochannel with Surface Roughness Characterized by Fractal Cantor

**DOI:** 10.3390/mi8060190

**Published:** 2017-06-19

**Authors:** Pengfei Lu, Xiangdong Liu, Chengbin Zhang

**Affiliations:** 1 Energy and Power Engineering, School of Hydraulic, Yangzhou University, Yangzhou 225127, Jiangsu, China; pfabc0826@126.com; 2 Key Laboratory of Energy Thermal Conversion and Control of Ministry of Education, School of Energy and Environment, Southeast University, Nanjing 210096, Jiangsu, China; cbzhang@seu.edu.cn

**Keywords:** electroosmotic flow, fractal, surface, roughness

## Abstract

Molecular dynamics simulation is applied to study the electroosmotic flow in rough nanochannels, with particular attention given to the fluid–solid interactions. In the simulation, the surface roughness is characterized by a fractal Cantor. The roles of roughness height and fractal dimension on nanoscale electroosmotic flow are examined and analyzed. The concentration distributions, zeta potential and electroosmotic velocity are presented and investigated. The results indicate that surface roughness plays a significant role in the fluid–solid interaction and nanoscale electroosmotic flow. The distribution of dipole angle for water molecules in both the near-wall region and middle region is almost unaffected by surface roughness; however, a significant difference of dipole angle distribution is observed in the fluid region away from the wall. Interestingly, the concentration distributions, electroosmotic velocity and zeta potential are highly affected by the surface fractal dimension, even with the same roughness height.

## 1. Introduction

With growing interests in micro/nano-fluidic applications (bio-MEMS, DNA identification and micro fuel cell, etc.), electrokinetic flows have become one of the most important microfluidic techniques [1,2,3,4,5,6,7,8,9,10]. In this field, electroosmotic flow has been widely used for mixing, pumping and separating in micro-devices, owing to its inherent advantage of having a simple structure, small size, controllability, reusability and no moving elements. Therefore, the understanding of electroosmotic flow in nanochannels is of significance for the research and development of new micro-devices.

Typically, a certain amount of charges appears on the solid surface and the counterions accumulated at the liquid–solid interface when an ionic solution comes into contact with the solid wall, which leads to the formation of an electric double layer (EDL). In the near-wall region, the fluid particles usually adhere to the solid surface and this is known as the Stern layer. The fluid flow outside the Stern layer is driven by the imposed electric field. This type of electrokinetic flow is known as electroosmotic flow. The strong fluid–wall interaction, which is observed in nanochannels, is one which is significantly different to that within macroscale channels. The classic theory of electroosmotic flow has been well developed and it could accurately predict the observed macroscopic phenomenon; however, the electroosmotic flow is a size-sensitive electrokinetics phenomenon [11] and the feasibility of electroosmotic models in detail is difficult to verify in nanoscaled channels. What is more, is that all solid surfaces are inevitably rough, even with the most advanced micro machining technology. Thus, it has become more difficult to fully understand the electroosmotic flow in rough nanochannels, and therefore, new experimental [12] and numerical methods have come to be urgently needed.

During the past decade, with the improvement of computers’ data processing and storage capacities, several numerical simulation methods have been successfully applied to study the micro/nano-scale electroosmotic flow [13,14,15,16]. Among these, the molecular dynamics simulation method has been shown to have advantage in this field. There have already been a series of studies on electroosmotic flow in rough nanochannels via the molecular dynamics method [13,17,18,19,20]. Qiao et al. [17] studied the nanoscale electroosmotic flow with consideration of molecular level surface roughness. They showed that the ion distribution and electroosmotic velocity is significantly altered by the rough surface when the thickness of the EDL is the same as the roughness height. Kim and Darve [18] investigated the electroosmotic flow in rough nanochannels with rectangular surface roughness and showed that the flow rate and zeta potential decrease with increasing roughness. Using extensive molecular dynamics simulations, Joly et al. [19] showed that zeta potential is greatly affected by the charged surface wettability. For the non-wetting surface, the zeta potential arising from electrokinetic effects is significantly amplified by the interfacial slippage. In these investigations, the geometric structures of rough surfaces are still modeled by regular patterns (such as rectangular and triangular roughness) [15,17,20,21,22,23]. In fact, there is no regular roughness on the surface. The surface morphology is usually sophisticated, and the multiscale and self-affine fractal behavior is exhibited for the surface roughness [24]. Recognizing the multiscale and self-affine properties, Chen et al. [25,26] have introduced the fractal method to describe the surface roughness in microfluidics and investigated their roles in micro/nano-scale fluid flow and heat transfer. This investigation demonstrated that the fractal dimension is of significance in microscale single flow, boundary slip and temperature jump at the fluid–solid interface.

Despite the previous studies on the electroosmotic flow with regular rough wall, or on the heat transfer by introducing fractal properties in wall roughness, however, whether or not the mass transfer of electroosmotic flow is related to the surface fractal properties (both the roughness height and the roughness fractal dimension) is not completely known. In particular, the fluid behavior near the liquid–solid interface with different fractal dimensions is necessary to be clarified. Therefore, in the current investigation, a molecular dynamics simulation is used to study the electroosmotic flow in a nanochannel with surface roughness as described by fractal Cantor. The effects of the surface roughness height, as well as the roughness fractal dimension, on the electroosmotic velocity and the zeta potential are investigated, and the detailed behaviors of fluid particles at the fractal rough wall are presented. We hope that the fractal method introduced in our work will provide valuable insights into the optimal design of microchannel surfaces for electroosmotic flow studies.

## 2. Fractal Description of Surface Roughness

**Figure 1:**
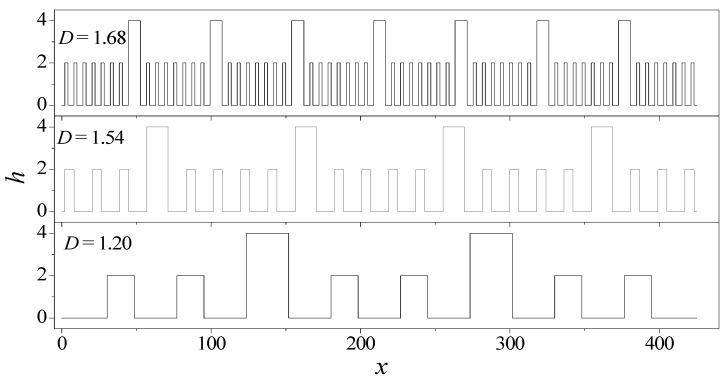
Fractal description of rough surface with different fractal dimensions.

The description of the roughness profile is the premise for understanding how the surface roughness affects the electroosmotic flow. The previous studies have demonstrated that the surface roughness possesses the fractal behaviors of multiscale and self-affine properties. To this end, the fractal Cantor is applied to describe the distribution of asperities on the surface. In the fractal Cantor, its roughness profile is constructed by joining the segments of a Cantor set (see Figure 1). The length and height of each level can be expressed as [27]:

(1) $$L_{n+1}=\left(\frac{1}{f_{z}}\right)L_{n}={\left(\frac{1}{f_{z}}\right)}^{n+1}L_{0}$$

and

(2) $$h_{n+1}=\left(\frac{1}{f_{z}}\right)h_{n}={\left(\frac{1}{f_{z}}\right)}^{n+1}h_{0}$$

where *L*_0_ is the nanochannel length, and *f_x_*, *f_z_* are the proportionality coefficients. In this fractal description, the 0th roughness height *h*_0_ = 2*δ* (*δ* denotes the root-mean-square roughness height), and the self-affine fractal dimension of roughness is calculated from [28]:

(3) $$\begin{array}{l} \phi =\sum_{bonds}\frac{k_{i}}{2}(l_{i}-l_{i,0}{)}^{2}+{\sum_{angles}\left(\theta_{i}-\theta_{i,0}\right)}^{2}+\sum_{torsions}\frac{V_{n}}{2}(1+\cos(n\omega -\gamma ))\\ \;+\sum_{i=1}^{N}\sum_{j=i+1}^{N}\left(D_{0}[{\left(\frac{\sigma_{ij}}{r_{ij}}\right)}^{9}-{\left(\frac{\sigma_{ij}}{r_{ij}}\right)}^{6}]+\frac{q_{i}q_{j}}{4\pi \varepsilon_{0}r_{ij}}\right) \end{array}$$

where *s* is the asperity number. The detailed specification of the roughness parameter in the construction of fractal Cantor is presented by the authors of [28,29].

In this study, the proportional coefficients are assumed to be *f_x_* = 13/9, *f_z_* = 2 in the generation of fractal Cantor. If the asperity number *s* = 3, 6, 8 are given, the fractal dimensions can be determined for these three surface roughness, which are *D* = 1.20, 1.54, 1.68, respectively. The profiles for these three rough surfaces are depicted in Figure 1. This plot provides a detailed analysis of the fractal characteristic of the rough surface. It is seen that the statistical roughness heights of the surface profile are identical, but the spectrum of the rough surface (i.e., fractal dimension) is evidently different. This result indicates that the fractal dimension is an independent variable in the description of the roughness profile.

## 3. Molecular Dynamics Simulation Details

Aiming to explore the rough surface effect on electroosmotic flow, this paper constructs a molecular level model of electrolyte-solution flow, driven by the imposed electric field in rough nanochannels. In the model, the rough surface is described by fractal Cantor on *xz* plane. The molecular dynamics method is applied to numerically solve the electroosmotic flow. In order to analyze the difference between the rough surface and smooth surface, the electroosmotic flow in smooth nanochannel is also simulated.

**Figure 2:**
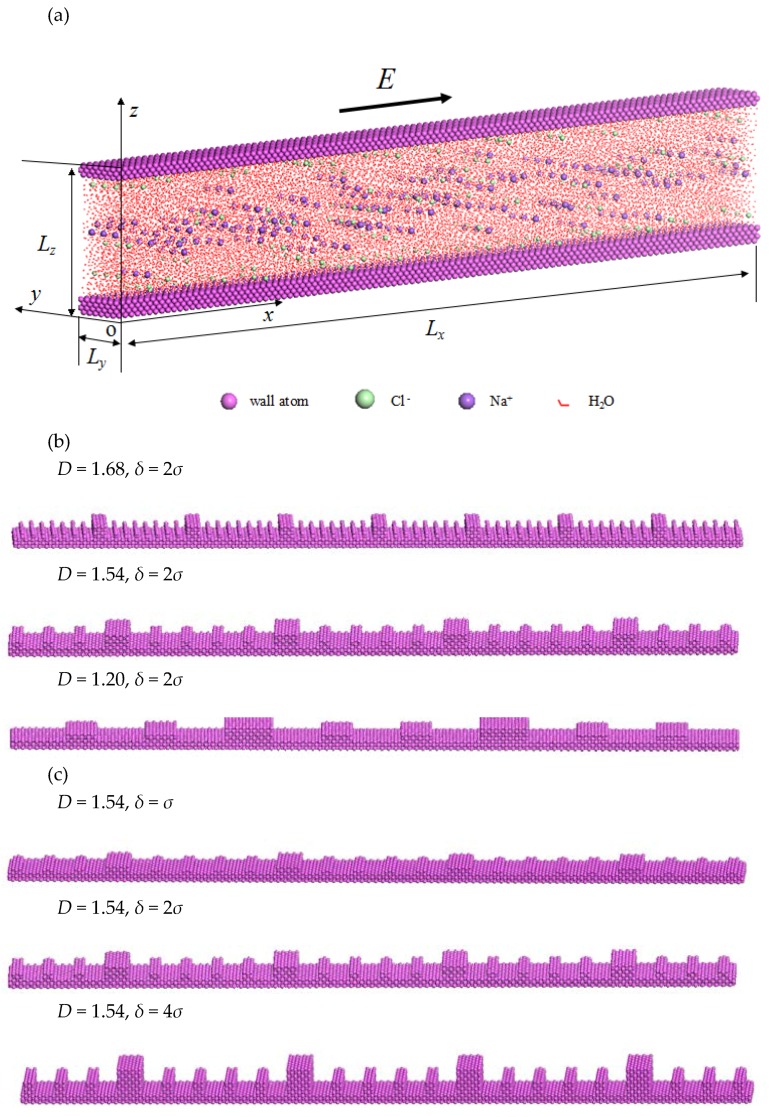
Electroosmotic flow in nanochannels: (**a**) Model of Electroosmotic flow; (**b**) Rough walls with different fractal dimension; (**c**) Rough walls with different rough height.

For the electroosmotic flow in the smooth nanochannel (see Figure 2a), the electrolyte solution is sandwiched between the lower and upper solid walls. The lower and upper solid walls are composed of three and four layers of solid atoms, respectively. All the solid atoms are arranged in the face centered cubic (FCC) unit cell. The dimensions of the whole computational domain are assumed to be *L_x_* = 42.52 nm, *L_y_* = 2.43 nm and *L_z_* = 7.31 nm. In the simulation, the NaCl solution is selected as the electrolyte solution which contains 24,000 water molecules, 120 Na^+^ ions and 240 Cl^−^ ions; the water molecules and ions are randomly distributed inside the nanospace. The density of the electrolyte solution is 1.3 g/cm^3^. The innermost layers of lower and upper solid walls have been assigned 120 discretely-distributed positive electric charges in total to balance the negative charges in the electrolyte solution. The electric field is imposed along *x* direction to drive the flow of electrolyte solution in the nanochannel.

For the electroosmotic flow in rough nanochannels, with other conditions remaining unchanged, the rough lower wall is modeled by adding extra solid atoms on the smooth wall to form a fractal roughness as described above. In the current study, two typical cases of rough surface are considered in order to analyze how the surface topography affects the electroosmotic flow. One case is where the fractal dimensions of rough walls are different, while the statistical roughness heights are identical (see Figure 2b). The other case is where the statistical roughness height is different, while the fractal dimension is the same (see Figure 2c).

In the molecular dynamic simulation, Newton’s Second Law is obeyed by the moving particles, i.e., Newton’s equation of motion is applied to characterize the particle motion of electrolyte solution in the nanochannel, which is expressed as [30]:

(4) $$m_{i}\frac{d^{2}\vec{r}}{dt^{2}}=\sum_{j\neq i,j=1}^{N}{\vec{F}}_{ij}+\sum_{j_{w}\neq i,j_{w}=1}^{N_{w}}{\vec{F}}_{ij_{w}}+F_{out}\vec{i}$$

where subscript *i* denotes the ith fluid particle. The three terms from left to right on the right-hand side of Equation (4) are the interaction force of fluid–fluid particle, the interaction force of fluid–solid particle, and the electric force. In the simulation system, there are *N* fluid particles (including water molecules, Na^+^ ions and Cl^−^ ions) and *N_w_* solid wall particles.

In the present work, COMPASS forcefield [31] is applied to deal with the intra- and inter-molecular interactions within the system. The functional form mainly contains five terms, i.e., bond stretching, angle bending, torsional, van der Waals and electrostatic terms, which can be written as:

(5) $$\begin{array}{l} \phi =\sum_{bonds}\frac{k_{i}}{2}(l_{i}-l_{i,0}{)}^{2}+{\sum_{angles}\left(\theta_{i}-\theta_{i,0}\right)}^{2}+\sum_{torsions}\frac{V_{n}}{2}(1+\cos(n\omega -\gamma ))\\ \;+\sum_{i=1}^{N}\sum_{j=i+1}^{N}\left(D_{0}[{\left(\frac{\sigma_{ij}}{r_{ij}}\right)}^{9}-{\left(\frac{\sigma_{ij}}{r_{ij}}\right)}^{6}]+\frac{q_{i}q_{j}}{4\pi \varepsilon_{0}r_{ij}}\right) \end{array}$$

where *l* denotes the bond length, *θ* denotes the bond angle, and *ω* denotes the dihedral angle. For two non-bonded interactions, *σ* and *D*_0_ are the equilibrium distance and well depth for LJ-96 potential, *r_ij_*, *q* and *ε*_0_ are the distance of arbitrary atom pairs, atom charge and dielectric constant in Coulomb potential. COMPASS forcefield is enabled to accurately predict the gas-phase and condensed-phase properties (conformational, structural and cohesive energies, etc.) in a wide range of molecules via the solution of Newtonian equation. To simulate the electroosmotic flow, the electric force is utilized to drive the electrolyte-solution flow in nanochannels. The Forcite module in Materials Studio software [32] is used for the whole simulation.

Starting from a random configuration, the above models were firstly relaxed by molecular mechanism method. Periodical boundary conditions were exerted along both the *x* and *y* directions. The steepest descent, adjusted on the basis of the set Newton-Raphson and quasi-Newtown methods, were employed to realize the rapid convergence. Both the van der Waals and Coulombic interaction have been calculated using Ewald summation (accuracy: 10^−3^ kcal/mol). Then, the equilibration and production processes (10^5^ steps for both two processes) were conducted under canonical ensemble (NVT). The fluid temperature inside the nanochannel was maintained at 300 K through the use of Nose thermostat [33]. The electrical field, which was imposed along the *x* direction, is assumed to be *E* = −1.0 V/nm. The distributions of concentration and velocity for the electrolyte solution across the nanochannel were determined by the use of the binning method.

## 4. Results

### 4.1. Distributions of Water Molecules and Ions

**Figure 3:**
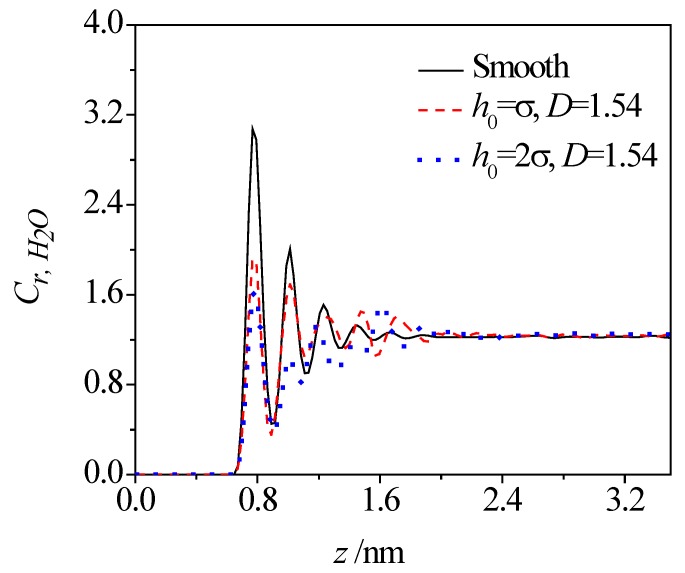
Effect of roughness height on concentration distribution of water molecules.

**Figure 4:**
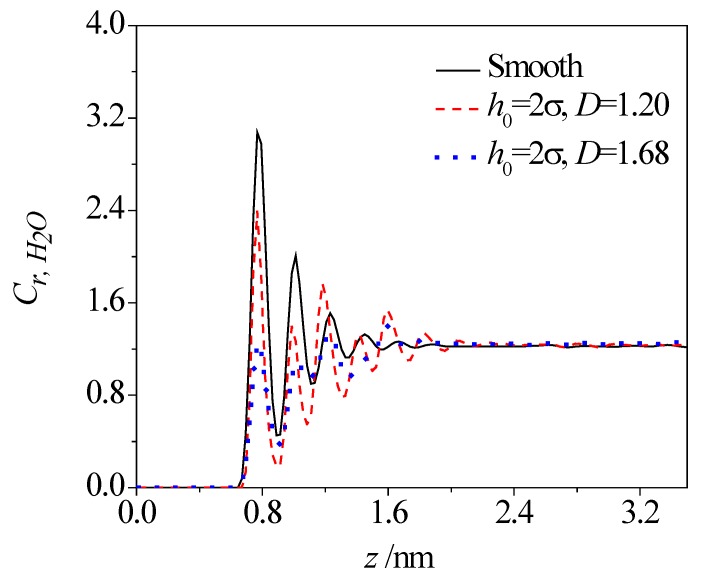
Effect of fractal dimension on concentration distribution of water molecules.

Figure 3 and Figure 4 present the concentration profiles of water molecules across the smooth and rough nanochannels. In the plot, the relative concentration of water, *C*_r, H_2_O_, is a ratio of local concentration within each slice to the mean concentration of the whole system. As shown, despite of the presence of roughness on the lower wall, the water molecules show the first peak at 0.3 nm away from the solid surface (thickness of the lower wall is about 0.54 nm) and then a weaker oscillation toward the nanochannel center. The layering distributions are significant until 1.5 nm away from the nanochannel surface. Compared with the smooth nanochannel, the first-peak and second-peak values of water concentration in a rough nanochannel decrease and become even lower than those in the bulk solution for the rough nanochannel. For given fractal dimensions, variations in roughness height triggers the redistribution of water molecules. Increases in roughness height contributes to the reduction of both the oscillation magnitude and peak value of water concentration. Even for a given roughness height, as the fractal dimension increases, the variation of the rough surface is more frequent, and narrower valleys are formed on the rough surface, which make the water molecules harder to access in the valley. In this case, the oscillation magnitude and peak value of water concentration are decreased for rough surfaces with a larger fractal dimension.

**Figure 5:**
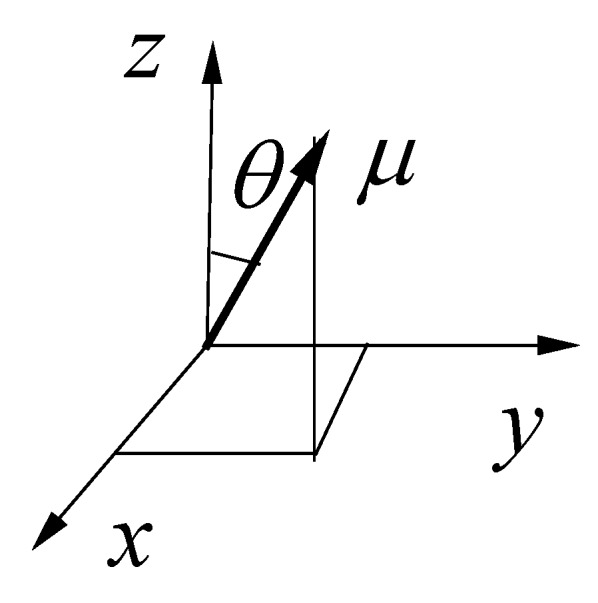
Schematic of dipole angle *θ*.

**Figure 6:**
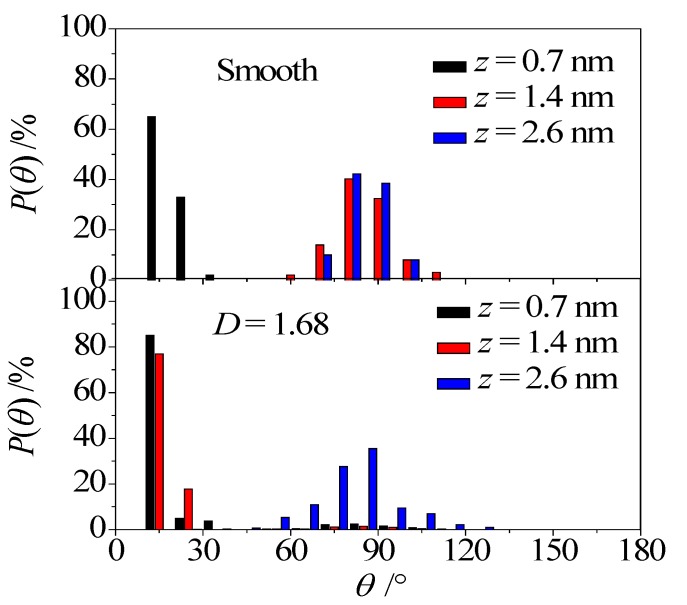
Probability density function of water molecule orientations.

Due to the bias shared electrons between the oxygen atom and the hydrogen atom in water molecules, the separation of charge (i.e., the dipole moment, *μ*) exists in the water molecules. The angle between the vector *μ* and *z* axis (see Figure 5), *θ*, is employed to analyze the water arrangement in the nanochannel. Based on the coordinates of H and O atoms in one water molecule, the angle (*θ*) between the vector *μ* of the water molecule and *z* axis could be obtained. By analyzing the number of water molecules whose dipole angle *θ* located at the certain regions from 0 to 180 degrees with the interval of 10 degree, the probability density distribution of the dipole angle *θ* for water molecules at different distances away from the lower wall could be gained, which is depicted in Figure 6. As shown in the figure, almost all of the water molecules in the near-wall region (*z* = 0.7 nm) have *θ* < 30°, no matter whether the wall is smooth or rough, indicating that the water molecules could adjust the oxygen atoms towards to the wall because of the predominant water–wall electrostatic interaction. In the fluid region away from the surface (for example, *z* = 1.4 nm), the distribution of water molecules dramatically varies in the smooth channel, in which the dipole angle lies between 60° and 120°. Differing from the smooth nanochannel, the water molecules away from the surface (*z* = 1.4 nm) in the rough nanochannel are still *θ* < 30°, and there is a small probability distribution at 60° < *θ* < 120°, indicating that the water molecules are attracted to convex side. When the water molecules are in the middle region of nanochannel (*z* = 2.6 nm), there is a Gaussian-like distribution for *θ* and the most probable angle is *θ* ≈ 90°, for both the smooth and rough nanochannels. This trend implies that most water molecules in the middle region prefer the orientation paralleled to the electric force direction. In summary, the distribution of dipole angle for water molecules in both the near-wall region and the middle region is almost unaffected by the surface roughness; however, a significant difference of dipole angle distribution is observed in the fluid region away from the wall.

**Figure 7:**
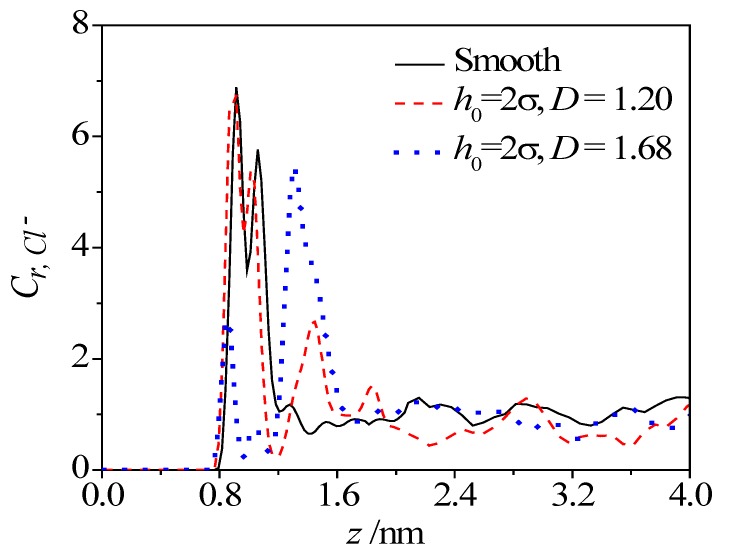
Effect of fractal dimension on concentration distribution of Cl^−^ ion.

To give a clearer understanding of the effect of roughness, Figure 7 compares the concentration profiles of Cl^−^ ion along *z* direction, in both the smooth nanochannel and the rough nanochannel. As shown, two close peaks of Cl^−^ concentration in the near-wall region appears at about 0.2 nm and 0.5 nm that is away from the smooth solid surface. The existence of roughness reduces these two peak values, but introduces an extra small peak at about 0.9 nm, that is away from the solid surface, which corresponds to the asperity position on the surface. As the region departs from the solid surface, the concentration of Cl^−^ ion quickly decreases to the bulk value. It is also seen from the figure that the first two peaks weaken and the extra small peak strengthens as the fractal dimension increases. When the dimension *D* increases to 1.68, the extra peak becomes much larger than the first two peaks. This trend means that the rough surface with higher fractal dimension could attract more Cl^−^ ions in the valley.

The distributions of water molecules and Cl^−^ ions, as stated above, can be understood through the analysis of the interaction between the water molecules, the ions and the wall atoms, at the molecular level. For water molecules near the smooth surface, the van der Waals force of water molecules and solid atoms dominates the orientation of water molecules and hence, leads to the first water layer. When contacted with the rough surface, the steric hindrance becomes non-ignorable for the water molecules trapped in the valley. The asperities on the surface affect the layering distribution of water molecules. The larger the fractal dimensions, the narrower the valleys on the surface, which results in a more significant steric effect. For Cl^−^ ions, the electrostatic force among the ions, wall atoms and external electric field is another important factor. On the rough surface, the positive wall atoms attract the Cl^−^ ions close to the wall and even in the valley, but the external electric force tries to make the ions escape from the valley. The steric effect of asperities hinders its motion. The radius of Cl^−^ ion is approximately 0.18 nm. What is more, the interaction between Cl^−^ ions and water molecules causes a hydration layer around the ion to form, due to the solvent effect. These factors make the ions harder to access in the narrower valley, as the fractal dimension of the rough surface increases. As a result, the first peak for the concentration in the rough nanochannel becomes smaller, which is similar to the case of water molecules. Caused by the densely-packed ions in the near-wall region as well as the strong electrostatic repulsion, more ions are harder to accommodate at the location of the first peak.

**Figure 8:**
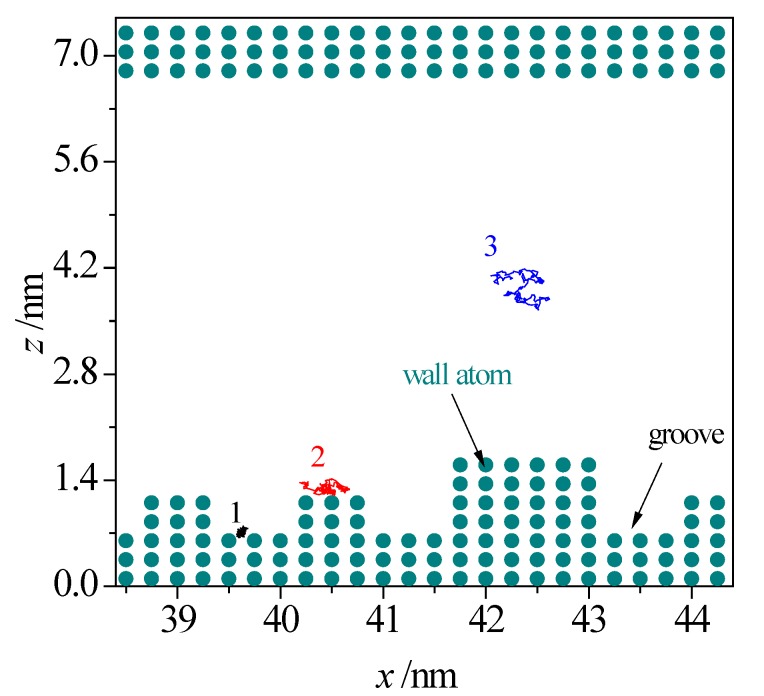
Motion trajectory of fluid atoms in a rough nanochannel.

To intuitively exhibit the influence of surface morphology on the electrolyte solutions, Figure 8 illustrates the typical motion trajectory of fluid atoms in the rough nanochannel, which is projected on the *xz* plane for a time span of 20 ps [34,35]. As shown in the figure, the fluid atoms in the middle region in nanochannel (e.g., atom 3) exhibits the superior motion ability, however, the motion range of fluid atoms close to the wall is very limited (e.g., 1 and 2). The fluid atoms in the valley are especially restricted on the solid wall (e.g., atom 1). The phenomenon suggests that the wall roughness could significantly reduce the motion ability of fluid atoms in the vicinity of the solid wall, that it should overcome the electrostatic force form the charged surface and that the extra hindrance from the asperities on the wall for the fluid atoms in the corner comes from when these are moving along the nanochannel.

### 4.2. Zeta Potential and Electroosmotic Velocity

The above discussions (in Section 4.1) suggest that the surface roughness of nanochannels with different roughness heights and fractal dimensions, could significantly alter the distributions and motions of fluid particles near the liquid–solid interface, which is similar to the effects of regular roughness [17,36]. However, how the zeta potential (*ζ*) [37] and electroosmotic velocity, the critical parameters in electroosmotic flows, are related to the surface morphology is still necessary to be clarified. Zeta potential is the potential at the edge of the Stern layer, and it follows the Helmholtz–Smoluchowski relation with the electroosmotic velocity of bulk solution in nanochannels, which is shown as:

(6) $$\zeta =-\frac{u_{a}\eta }{\varepsilon E}$$

where *u_a_* represents the electroosmotic velocity beyond the EDL in nanochannel, *η* represents the viscosity [38,39], *E* represents the applied external electric field, *ε* represents the permittivity of bulk electrolyte solution (*ε* = *ε*_0_*ε*_r_, where *ε*_0_ is the vacuum permittivity and *ε*_r_ is the dielectric constant of the bulk electrolyte solution).

**Figure 9:**
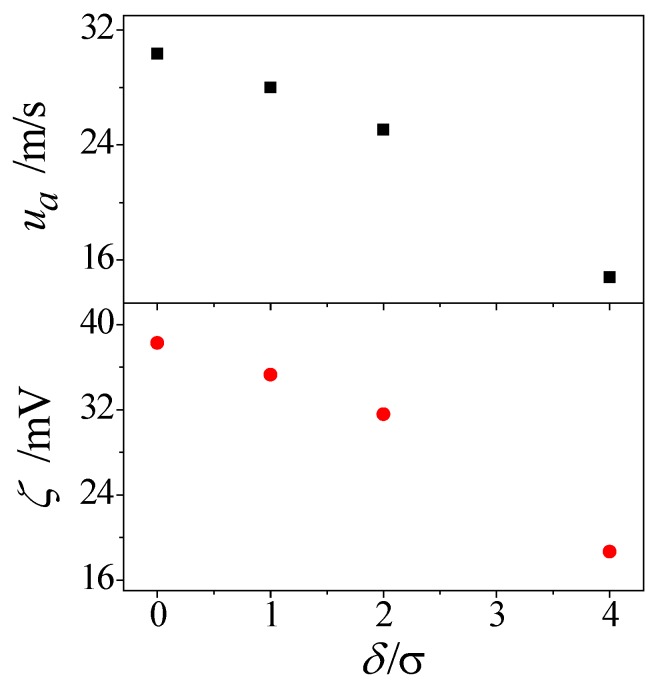
Effect of roughness height on electroosmotic flow.

In a nanochannel, the electroosmotic flow is directly related to the microscopic behavior of electrolyte solution at fluid–solid interfaces. The solid wall induces order in the adjacent water molecules as well as the Cl^−^ and Na^+^ ions, which in turn controls the degree of fluid–solid interaction and finally affects the bulk velocity. Figure 9 presents the effect of roughness height on the zeta potential and electroosmotic velocity. As shown, the presence of roughness reduces the zeta potential and electroosmotic velocity. In addition, both these two evaluation parameters decrease monotonously with increasing roughness height. This is mainly attributed to the fact that the fluid particles find it difficult to get access into the bulk flow in the nanochannel with a larger roughness height.

**Figure 10:**
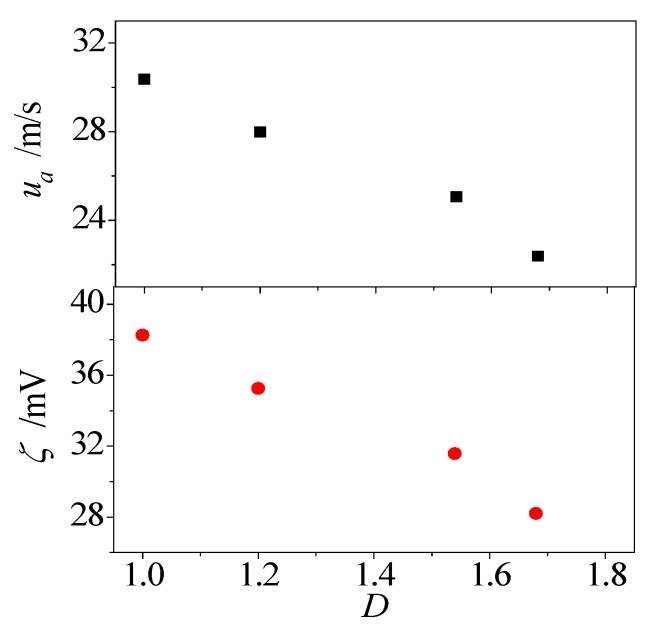
Effect of fractal dimension on electroosmotic flow.

Apart from the roughness height, the nanoscale Electroosmotic flow is also affected by the fractal dimension of surface roughness, as shown in Figure 10. As expected, the electroosmotic velocity and zeta potential are influenced by the fractal dimension of surface roughness. Increases in the fractal dimension bring more and narrower valleys and asperities on the solid surface, and provide a larger area for fluid-solid interaction, which brings significant confinement for the fluid particles in valleys, and greatly restricts the motion capability of fluid particles close to the solid surface.

## 5. Conclusions

In this paper, we constructed a molecular level model of electroosmotic flow in rough nanochannel with surface roughness as described by fractal Cantor. The molecular dynamics simulations were carried out to investigate the electroosmotic flow in a nanochannel. The role of surface topography on the water molecule and ion distributions, zeta potential and electroosmotic velocity were examined and analyzed. Based on the current investigation, the following conclusions were obtained:

(1) The presence of surface roughness reduces the concentration oscillation magnitude of water molecule close to the solid wall. In addition, the oscillation magnitude of water concentration is decreased by both the roughness height and fractal dimension.
(2) Almost all of the water molecules in the near-wall region have a dipole angle *θ* < 30° no matter whether the wall is smooth or rough. For the region away from the surface, the dipole angle lies between 60° and 120° for water molecules in the smooth channel, however, the dipole angle of water molecules in the rough nanochannel are still *θ* < 30°. For the water molecules in the middle region, there is a Gaussian-like distribution for dipole angle *θ* and the most probable angle is *θ* ≈ 90° irrespective of nanochannel surface condition.
(3) The surface topography plays a significant role in the fluid–solid interactions and electroosmotic flow. The existence of roughness contributes to the reduction of the electroosmotic velocity and zeta potential, and they decrease monotonously with increasing roughness height. Interestingly, the electroosmotic velocity and zeta potential also highly affected by the surface fractal dimension, even with the same roughness height.
